# A Detailed Protocol to Enable Safe-Handling, Preemptive Detection, and Systematic Surveillance of Rat-Vectored Pathogens in the Urban Environment

**DOI:** 10.3389/fpubh.2016.00132

**Published:** 2016-07-14

**Authors:** Michael H. Parsons, Ronald J. Sarno, Michael A. Deutsch

**Affiliations:** ^1^Department of Biology, Hofstra University, Hempstead, NY, USA; ^2^Medical Entomology, Arrow Exterminating Company, Lynbrook, NY, USA

**Keywords:** arthropod vectors, disease emergence, pathogen surveillance, *Rattus norvegicus*, over-crowdedness, RFID, rodentology, urban ecology

## Abstract

We detail a five-stage protocol to address physical barriers and experimental limitations that have hindered routine pathogen monitoring of wild rats in urban settings. New York City potentially harbors from 2 to 32 million rats among its 8-million people. However, at a time, when people are most vulnerable to disease from over-crowdedness brought on by increased urbanization of society, the difficulty of studying wild rats has led to a paucity of ecological and epidemiological research. Challenges of safely handling animals and the difficulties of identifying individual animals and the emergence of their respective pathogen loads (timing of infection) have impeded progress. We previously reported a method using radio frequency identification paired with load cell and camera traps to enable the identification of individual animals and subsequent monitoring of the animals’ weights (an indicator of health). However, efficient pathogen surveillance requires repeated captures of the same individual in order to isolate and document the emergence of new pathogens, or variations in pathogen load, over time. Most of these barriers are now addressed in our protocol, which is aided by the use of a mobile, outdoor laboratory, followed by incorporation of pheromone-based lures to attract individuals back to active sensors, within a camera trap. This approach allows for the assessment of individual animal health, behaviors under camera, and changing pathogen loads and weights in most urban environments (e.g., financial district, docks, sewers, and residential). Five phases are described and presented: (1) site selection and urban trapping, (2) anesthetization, (3) serological and ectoparasite collection, (4) microchip implantation, and (5) retrapping and luring animals back to active remote sensors. In order to fulfill the unmet call for preemptive pathogen surveillance, public health officials and researchers may wish to adapt, or modify, similar protocols to ensure early detection and monitoring of rat-borne zoonoses, before they become problematic.

## Introduction

Seventy-five percent of the world’s human population is expected to live in urban settings by 2050. With the average rate of population growth in urban cities almost twice as high as the overall growth rate (1), people are becoming more densely crowded in smaller spaces where they are more vulnerable to fires, diseases from fouled water and food supplies, and insects and other arthropods that thrive in city spaces (2). The primary vector of many hazards that urban people encounter is directly, or indirectly, related to urban rodents. As urban commensal rats, rats are associated with human population density, sanitation, and hygiene, and as we grow in number and production of refuse, their numbers proliferate. These species cost the U.S. economy $19 billion per year from food loss, infrastructure damage, and disease (3). As urbanization continues, it is essential that ecological monitoring and preemptive disease–pathogen surveillance become routine, robust, and spatially replicated. Yet, the difficulties of studying rodents in the urban environment have thwarted ecologists and health professionals for decades (4), despite our density-dependent vulnerabilities (2) and strong calls for preemptive pathogen surveillance (5).

It would be inappropriate to address the physical barriers to research without first acknowledging the complex social issues that influence urban rat studies. Rats are associated with filth, disease, and poverty (6) and are often vilified in the popular culture. Infestation can lead to heavy fines, damaged reputations, and even the closing of commercial business by municipal authorities. Conversely, homeowners and especially businesses may be less likely to share knowledge about their rat infestations with researchers who urgently need to gain access to these potential study sites in order to document the extent of infestation along with risks of infection and disease. The unique challenge of finding accessible urban study sites has been depicted by the ironic, but familiar, phrase “in a city of 32 million rats, no one *has any*.”

Conversely, it has been suggested that in New York City, a population exceeding 8 million people, there are fewer than 10 institutional researchers actively pursuing urban rat research (Corrigan, personal communication, New York City Department Health and Mental Hygiene). Therefore, the social constraints to studying wild city rats are even more daunting because they are magnified by several physical challenges.

Urban rodents are elusive, subterranean, and often go unseen, making knowledge about them prohibitively difficult to ascertain. It is common knowledge that rats detected during daylight hours imply that unnaturally high populations already persist in the immediate environment. Yet, most perceptions about rats are based on a disproportionately few individuals that are detected in the daytime in public, leading to over-generalizations based on more gregarious animals or migrating individuals. The vast majority of rats are not seen by human eyes. The non-independence of most observations leads to misinformation that is propagated by anecdotal accounts, hearsay, and the media. The primary means to combat this growing problem is to overcome the barriers necessary to study urban rats *in situ*, in their normal environment, replicated at the level of the individual animal.

Remote sensing with common tools, such as radio-telemetry or GPS, is often used to identify individual animals, and thus, ensure independence of a randomly selected sample size (7). Unfortunately, in order to affix remote sensors, rats need to be trapped, handled, and sedated. Wild rodents are heartier and more resilient than lab rats, often requiring higher doses of sedative for longer durations (Urshuula Dulakia, Institute Comparative Medicine, Columbia University). Additionally, these animals harbor a number of diseases, including blood-borne pathogens that can be transmitted *via* bites or scratches [*Streptobacillus*, rat bite fever (8)]. Researchers working with high densities of rodents may also come into contact with bacteria carried in urine and feces [*Leptospira, Salmonella*, or *Toxoplasma* (9)]. Rats also carry insect (fleas) and arachnid ectoparasites (ticks, mites) that vector other pathogens (10), such as *Borrelia* [Lyme disease (11)], *Rickettsia* [Rocky Mountain Spotted Fever (2)], or *Bartonella* [Cat-scratch disease (2)]. These problems are compounded in the built environment where satellite link-ups and radio signal are blocked by infrastructure and cannot persist uninterrupted between transmitter and sensor. As a result of these challenges, extensive gaps pertaining to rat ecology and disease surveillance have accrued within the scientific literature (12, 13).

Feng and Himsworth (4), Banks and Hughes (13), and Parsons et al. (7) have recently identified a few of the most prominent knowledge gaps. These include behavioral and ecological studies that address the natural causes of rat mortality, how rats interact with specific features of the built environment, and importantly, how rodent ecology influences disease–vector potential to humans. According to Firth et al. (5), we have only scant indication of the specific organisms that rats harbor deep within our city (10), or how these organisms are vectored throughout the city, or when new diseases emerge. Because so little preemptive monitoring has been done, Firth et al. (5) identified 18 potential pathogens completely unknown to science when sampling only 133 rats in New York City.

With public health on the line and humans only living more densely packed together in the coming decades, this begs the question as to how many more pathogens will be discovered once rodents are more regularly monitored? And what are the benefits of understanding pathogen toxicity and exposure *prior* to breakouts?

We have recently learned that rodent scents are effective (7) in recruiting microchipped animals back to active sensors that activate microchips in order to record individual identification on a connected data logger. Not only did animals returned to the location for additional observations under camera [some visited the sensor as often as 33 times in a single day (7)], additional recaptures allowed repeated measurements of the same individuals, where routine weight recordings and changes in pathogen load could be assessed.

We have provided a few solutions to some of the barriers prohibiting urban rat research. Thus, by offering our five-step protocol, our objectives are to encourage and enable researchers to more easily undertake systematic pathogen surveillance of rodent-borne pathogens in the built environment. We are now detailing this protocol along with an assay for ectoparasite removal, anticipated results based on the recent literature and consultation with local experts, and a brief discussion of the social and research-based implications inherent in these activities.

## Materials and Equipments

### Mobile Workstation

Stainless steel lab cart (flat top required)
Lab bench liner
Digital scale
Chlorhexidene
Betadine
Vetbond
Alcohol preps (200)
Calipers
Wahl cordless razor
Nalgene lab notebooks
Qwik Stop^®^ syptic powder

### Instruments

Sterile, razor-sharp surgical scissors
7.5″ utility scissors
Metal ectoparasite “nit” comb
Square grid Petri dishes
8″ Pyrex roasting pan
Dry ice
Heat bead sterilizer
Ear punch w/tags (optional)
Biohazard bags
Body fluid swabs (Swabs)
DNA collection tubes
DNA preservative

### Trapping

Safeguard^®^ rear-release animal traps #52818 (18″ × 5″ × 5″)
Detex BLOX (non-toxic fluorescent) (optional)
Trap catch emitters (×12)
Hydrogel packs (box)
Dark, standard sized pillow cases (×4)

### Anesthesia

30% Isoflurene (250 ml bottles) (×3)
USP-grade oxygen refillable e-cylinder w/regulator
Oxygen tank cart
Induction chamber
Matrix VIP 3000 (or similar) vaporizer
Flow meter
Scavenger system
Nose cone (rodent/feline mask)

### Field Gear (RFID)

Reader/antenna/SCU/control block (×2)
12 mm chips (30) + retractors (×3)
Pheromones (pooled scents from lab rats)

### Camera Traps

Cameras ScoutGuard SG550V (×6)
Batteries AA Panasonic (×3)
Memory SD (×12)
Security locks

### Personal Protective Equipment

Hard hat/vests
N95 masks, or charcoal masks for organic agents
Disposable gloves
Kevlar gloves
Hand sanitizer

## Stepwise Procedures

### Site Location, Prebaiting, and Trapping

Study sites should be identified from a combination of city blocks, public park spaces, and/or residential areas. Researchers should be prepared to extensively search for appropriate research sites. The most usual site configuration includes moderate-to-high rat-infested areas (whether rats are observed during daylight hours) in potentially underserved and potentially dangerous areas of the city. Factors to include are the level of infestation, researcher safety, and ability to undertake research in highly populated areas, but without public knowledge of activities. This discretion will help ease the social anxiety of property owners and willingness to share their properties. This information can be determined by calling the local health department or by assessment of “look listen and learn” protocol, commonly used by pest-management professionals. See Figure S1 in Supplementary Material for a printable, distributable depiction of this information.

(1) Trapping. Common knowledge suggests rats should be trapped using local foods that rats have previously become accustomed to. However, foods do not often result in “retrapping of the same animal” that may learn to associate danger with a food item, and food baits alone could be biased toward males (14). Rats, however, are less likely to habituate to scents from conspecifics. Therefore, attract animals into traps baited with mixed, pooled scents from conspecifics. A sufficient mixture of a range of pheromones will equally attract males, females, and juveniles (7, 15).
(2) Rats often travel “blindly,” using their guard hairs and vibrissae in contact with one or more surfaces to guard them. A high-lumen flashlight with ultraviolet (365 nm) backlight (such as the Nitecore^®^ P20UV) will help expose chemically marked areas where rats repeatedly touch their guard hairs to the wall, while navigating blindly. This leads to sebum pheromone trails and also microdroplets of urine. These marks glow blue–white if fresh or yellow–white if old (96 h or longer). Therefore, trap strategically. Place approximately five rear-door release, small mammal traps such as the Safeguard^®^ 18 × 5 × 5 model #52818, spaced a minimum of 1 m apart, close to the one or more edges of a wall, for every suspected colony assessed.
(3) Bait traps with fresh mixed scents obtained from soiled rat bedding, pooled from multiple male and female rats. It is common to use rats from a pet store or university-owned lab rats. Some care should be taken to minimize the age of the scent. To be safe, the scents should not be more than 48 h old when commencing the study.
  (3.1) In order to minimize further aging of pheromones, store scents in an airtight bag under minimal headspace (minimal air-pocket), ensuring the bag is closed tightly and stored in a cool, dry place.
  (3.2) Install alert-sensors (emitters) into the trap (widely available on the Internet) along with hydrogel (food and water packs), so that an alert will be transmitted by cell phone to the principal researchers, once an animal has been caught. Rats should never be left alone for longer than 8 h in a trap. Not only is this inhumane but a stressed animal will take longer to anesthetize and may develop infection, rendering it unlikely to survive long enough for data collection.
(4) Following captures, cover active traps with a dark pillowcase or neoprene cover to minimize the startle response when picked up and transported by humans.

### Anesthetizing and Safe-Handling Rodents

Eliminate many of the hazards of rats by following a safe-handling protocol.

**Figure 1:**
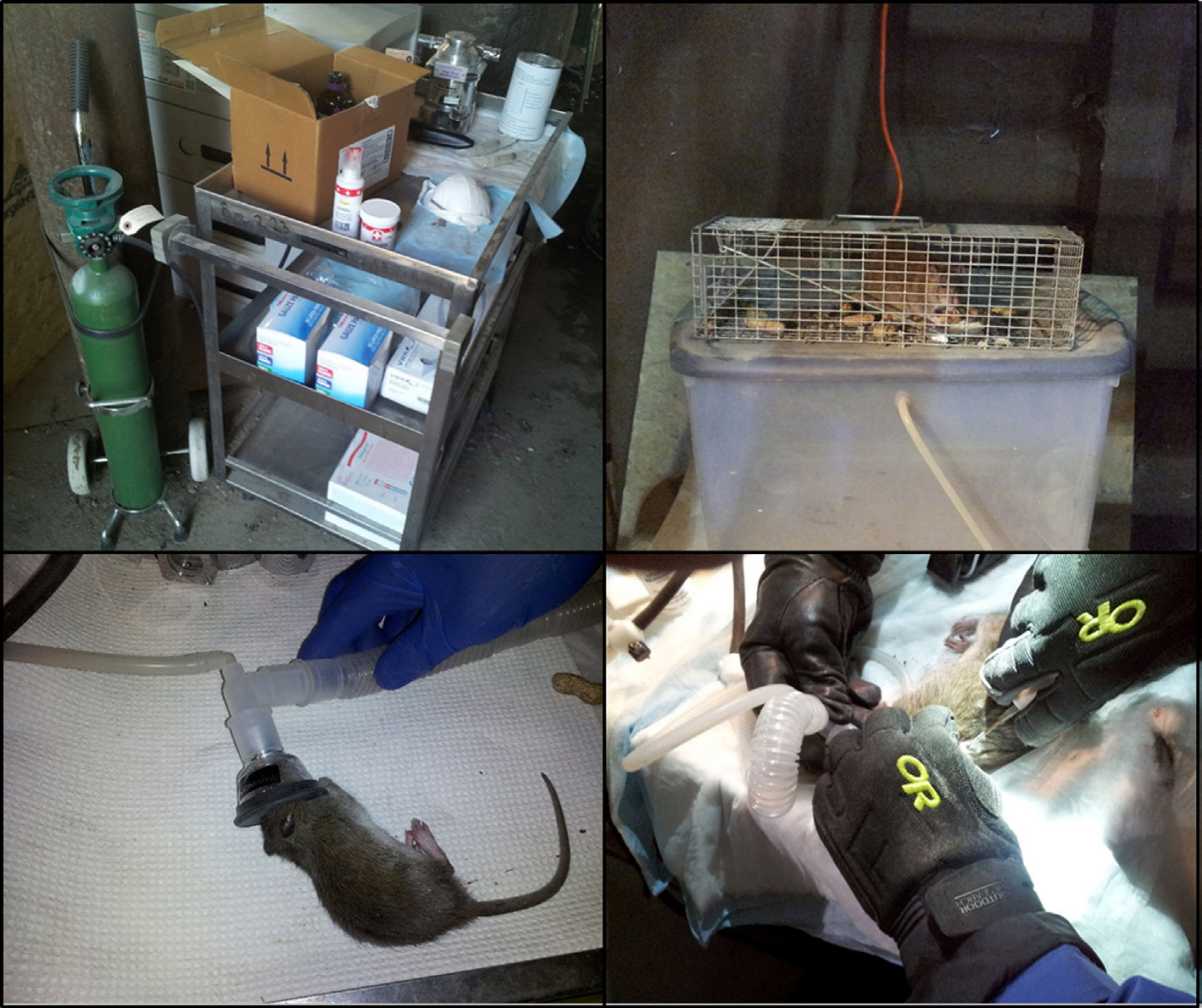
**(A–D)** Clockwise from top-left panel. Mobile indoor/outdoor mobile laboratory. Vaporizor and surgical trap with a dual-circuit flow system. **(B)** Induction chamber. Modified 61 cm × 91 cm oversized Tupperware container. **(C)** Feline mask and surgical tray. Mask affixed to rat with a strong seal. **(D)** Microchip implant. Pinch skin between fingers to form a small tent. Insert needle bevel up and eject.

(1) For ease of mobility across a wide range of urban environments, prepare a mobile indoor/outdoor lab that can be transported to multiple study sites. A stainless steel lab cart covered with lab bench liner (Figure 1A) will serve as storage, workspace, and surgical table for microchip implants. The table should house an isoflurane vaporizer (such as the Midmark Matric VIP 3000) with dual procedure circuit to distribute 30% isoflurane, and a scavenger system to collect CO_2_ wastes. One end should be attached to an induction chamber (an oversized 61 cm × 91 cm Tupperware container can be modified by cutting holes into the side; Figure 1B), with the second end attached to a feline surgical mask, such as those provided by VetEquip (Pleasanton, CA, USA). The size of the induction chamber should be large enough to easily hold the 18 × 5 × 5 rat trap, while being small enough to minimize the amount of isofurane required to fill the interior.
(2) Pre-workup (5–10 min)
  (2.1) At least two researchers will need personal protective equipment (PPE; masks, gauntlets), before beginning. One worker will be required to maintain hold on the animal, while the other adjusts the isoflurane flow, oxygen flow, and mask.
  (2.2) Sterilize table, check hoses, and connections for isoflurane unit. Isoflurane unit is on, all tubes connected. Table is clean. Instruments, microchips, and collection tubes on table.
  (2.3) Scan a selected microchip (Trovan^®^ 12 mm chip is appropriate) to provide initial data point.
  (2.4) Place dry ice on two Pyrex 8″-roasting pans. One for ectorparasite collection and the other for body fluid collections. Anal swab (Swubes^®^) should be accessible.
  (2.5) Update water-proof lab notebook hanging nearby.
  (2.6) Initially, set isoflurane to 5% with an oxygen flow rate of 2–3 ml O_2_/min to flow into the Tupperware induction chamber.
(3) Animal handling on surgical table (15–20 min)
  (3.1) Make way to trapped animal carrying a dark pillow case that will be used to place over cage, ideally, prior to animal spotting human presence. Gently transport the covered cage to the modified Tupperware induction chamber, while minimizing any anthropogenic noise (Figure 1B).
  (3.2) Dip entire locked cage into chamber for a minimum of 15 min until animal shows signs of slowed movement (partly sedated).
  (3.3) Open induction chamber with one researcher lifting the cage up at a 45° angle and released the rear release trap door gently freeing the animal onto the base on the chamber. The second researcher will use the toe-pinch method to determine level of sedation.
  (3.4) While the animal is mostly unconscious, use the claw method of a gloved hand (first two fingers over the shoulders, thumb and fourth finger gently constricting the diaphragm) and firmly lift the animal out of the cage and place onto the surgical area.
(4) Animal physical examination
  (4.1) The feline/rodent mask should be immediately placed over the animal, completely covering the nostrils and mouth (Figure 1C), regularly checking to make sure a good seal has been obtained. Despite semiconsciousness, it is common at this stage for the animal to kick and dislodge the mask.
  (4.2) The second researcher will reset the flow meter to the second part of the dual circuit (mask) and switch off petcock to the induction chamber. Ether flow should be re-adjusted to 5% isoflurane and 2–3 ml/min O_2._
  (4.3) Within 2–5 min, the animal should now be completely sedated. The physical characterization may now commence.
  (4.4) At this stage, the first researcher can open the lab notebook and place microchip identification (usually a sticker) in the appropriate lab entry form. Record sex, length, weight, and notes on appearance: porphyrin stains around head area, mottled or greasy appearance of hair, evidence of wounds or fighting, and general health (any breaks) of guard hairs and vibrissae.
  (4.5) With the mask still in place, the animal may be placed ventral side down, take sharp surgical scissors and snip 2.5–5 mm from end of tail, add to a marked DNA collection tube filled with preservative.
  (4.6) Save marked tube for transportation back to laboratory.

### Serological and Ectoparasite Collection

Body fluid and fecal samples for blood-borne and systemic bacterial and viral pathogens (5) can be collected, along with ectoparasites and their pathogens (10). The type and volume of fluid drawn will determine the variety of pathogens that may be assayed.

(1) Collect rectal swab, seal, and set aside to return to lab.
(2) Blood can be collected from the tail by squeezing/massaging the area around the DNA clipping into a capillary tube, or by cannulation of the lateral tail vain.
(3) Pressure and Kwik-stop styptic powder can be used to cauterize tail in case of bleeding.
(4) Ectoparasite collection
  (4.1) Place animal ventral side down. Grasp animal by abdominal and thoracic region. Forceps can be used to lift fur, while stainless steel nit/louse comb to brush animals’ hair both directions over square Petri dishes filled with dry ice (10).
  (4.2) Ectoparasites can be sorted visually, stored on dry ice, and transferred to −80°C for storage, until further analyses can be performed.
(5) Animals are now ready for pathogen detection assays.

Serological and tissue-based analyses of bacteria and viruses common to rodents in Manhattan are well described (5). Targeted molecular analyses (PCR-based assays) can be used to detect known human pathogens with published primers, such as *Campylobacter coli, Listeria monocytogenes, Rickettsia* spp. *Toxoplasma gondii, Vibrio vulnificus*, or *Yersinia pestis*, along with viruses, such as hantaviruses. Unbiased high throughput sequencing (UHTS) can be used on blood and rectal swabs to detect novel viruses not yet known to cause human disease. Frye et al. (10) has documented the steps necessary for DNA extraction from ectoparasites using DNeasy Blood and Tissue Kit (Qiagen Inc., Alameda, CA, USA), followed by PCR analyses.

### Microchipping Rodents

Most rats from 80 g and above can readily accommodate a 12-mm Trovan^®^ passive chip, roughly the size of a grain of rice.

(1) With animal on the table, dorsal-side up, use a battery-operated razor to shave 2 cm × 2 cm area of hair between the shoulder scapulae, making sure not to burn animal with the razor. Use alcohol or betadine and cotton swab with an inside out circling motion to sterilize shaved area.
(2) Pinch skin between fingers to form a tent. Insert needle bevel up, eject chip from retractor (Figure 1D), fingers in place to touch newly injected chip through outside of skin. Remove needle with a 180° twist to minimize bleeding.
(3) Check for bleeding, use gauze, firm pressure, and veterinary glue if necessary
(4) Remove mask and return animal to cage. Place cage near exact spot where it was trapped. Once the animal is moving again (usually 5–10 min, the animal is then ready for release), open door and let animal move out of cage.

### RFID and Pheromone Lures

**Figure 2:**
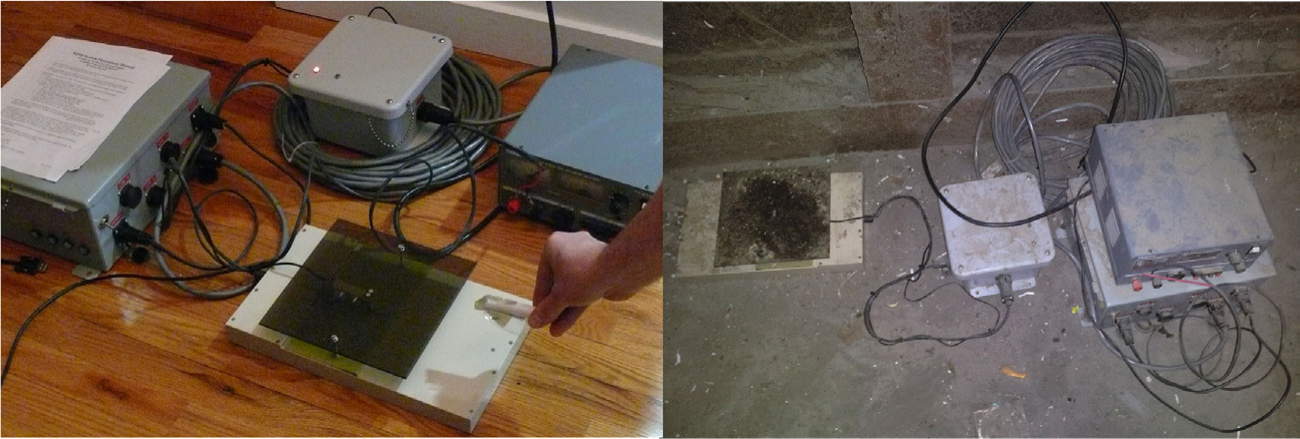
**RFID system**. Sensor, data logger, system control unit.

The radio-frequency identification (RFID) system will be preassembled in a place within 10 m of rat activity. We used the SA-148, SQID customizable PIT tag system (Seabird model; Figure 2) modified for this project by VANTRO systems, LLC. The unit was modified with a battery eliminator so that it could be powered by 240 V A/C mains. Data were captured by a data logger with a 2 GB memory capacity and USB connection. A load cell (38.64 ticks/g) was attached to the system control unit. Data were extracted from the system control unit through the use of Windows-based hyperterminal software. The system had an operating range of 0–100°F and remained powered at all times, except otherwise noted. The extracted data were then processed using a macro-enabled spreadsheet template (Microsoft Excel 2000) that was developed specifically for the RFID data. For the camera trap, we used a Wildgame Innovations^®^, Razor, 6.0-Megapixel Digital Trail Scouting mounted 2 m from the RFID reader and aimed ground-level at the antenna.

Used rat bedding can be collected from a pet store or university housing for lab rats, making sure five or more adult males and females are represented.

(1) Ensure RFID unit is powered on and that camera trap is switched to video mode.
(2) Set 100 g of fresh rat bedding on sensors

### Post-Workup (5–10 min)

(1) Check around all other traps for signs of rat activity (Figure S1 in Supplementary Material).
(2) Shut down mobile theater, wipe down table, remove instruments (place scissors in bead sterilizer), replace lab bench lining.
(3) Update lab manual
(4) Re-bait stations with 100 g fresh pheromones (both stations and sensors will have pheromones).
(5) Transport labeled collection vials (DNA, plasma, parasites) on dry ice to −80°-freezer.

### Continued Surveillance and Site Rotation

Rats are among the most sensitive animals to the smell of conspecifics. Similar to many mammals, they approach scents in a process called “scent inspection,” whereby most scents (even repellents) causes the animal to approach scents closely to ascertain their biological meaning (16). Thus, scents are the primary tool by which rats are repeatedly trapped, despite their proclivities to neophobia and habituation.

(1) Traps can be re-baited continually as long as their participation is required, in order to obtain adequate sample size and sufficient spatial replication.

### Statistics

Pairwise comparisons of visitation or dwell times can be performed between different pheromone types × sex using PROC ANOVA with the Tukey option in SAS v. 9.4 (Cary, NC, USA). Scatter plot of number of visits per animal expressed as days elapsed since first capture. This graph can be generated using the PROC SCATTER. Points may be jittered on the *y*-axis to visually represent multiple visits per day.

## Anticipated Results

**Figure 3:**
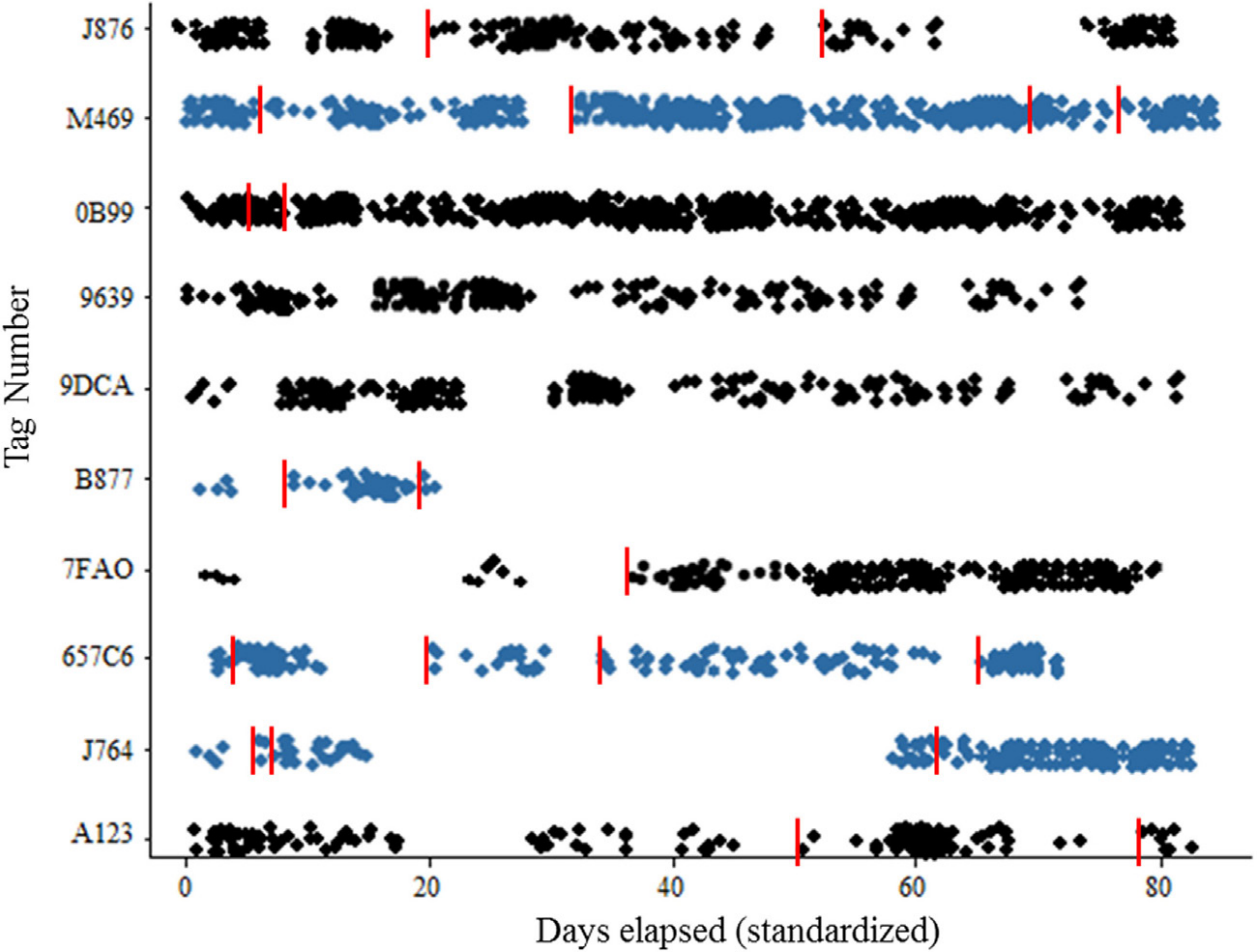
**Scatterplot of recruitment for wild Norway rats, *Rattus norvegicus* to sensor by sex**. Females represented by blue color, recaptures are indicated with vertical red line. Target population should be 20 animals × 7 sites = 140 marked individuals.

The downloads from RFID data loggers will indicate the time of day (time stamp), dwell times, frequency of visitation, weight, and all demographic factors for each individual animal over the duration of the study (Table 1; Figure 3). This information should then be linked to the emergence of any new pathogens, all rat recaptures (Figure 3), and any consequent changes in pathogen load (e.g., some species could become more or less prevalent; see example, data in representative Table 2). Information on individual behaviors will determine the association of sex, age, and other demographic factors on differing attractants such as specific types of pheromones (e.g., sebum, dander, urine, fecal, porphyrin, and saliva) (Table 3) or individual chemical constituents within pheromones. Females are demonstrated as visiting pheromones more frequently than males, but having similar dwell times.

**Table 1:** **Representative physical characterization**.

Tag #	Sex/juvenile	Site/location (a–g)	Weight begin (g)	Length (mm)	Health (1–4)	Marks/wounds	Visits to sensor (#)	Recaptures (#)	Weight change (g)
J876	M	b	448	25	3	Scarring/right flank	1032	3	20
M469	F	d	350	21	4		490	7	120
0B99	M	a	610	25	3	Mottled coat	1120	2	−10
9639	M	g	380	23	2	3.5 mm left dorsal	886	0	100
9DCA	M	g	224	22	2	Tail lost (8 mm remains)	429	0	275
B877	F	a	389	24	3	Porphyrin excess	0	2	111
7FAO	M/j	a	202	14	4		1420	1	340
657C6	F	d	490	20	3	Damaged vibrissae	630	7	−15
J764	F/j	c	96	15	4		553	3	156
A123	M	e	521	29	1	Left eye blind	632	4	5
**Average**									
Male	6		398	23				3	122
Female	4		331	20				5	93

*Example demographics and heath indicators of 10 wild, urban rats, *Rattus norvegicus*, monitored over 6 months. Target population should be 20 rats × 7 sites = 140 marked individuals. Sites should include financial district, public parks, and residential areas, where humans come into contact (directly or indirectly) with rodents*.

**Table 2:** **Anticipated results**.

Tag #	Sex/juvenile	Site captured (a–g)	Pathogens total(*genus*)	Ectoparasite vectored (*genus*)	Δ Winter	Δ Spring	Δ Summer
J876	M	b	Parvovirus; *Bartonella*; *Borrelia*	*Bartonella*; *Borrelia*	Parvovirus; *Borrelia*	*Bartonella*	
M469	F	d	*Y. enterocolitica*; *Bartonella*	*Bartonella*	*Y. enterocolitica*	*Bartonella*	
0B99	M	a	*Orbivirus*; *Rickettsia*				*Orbivirus*; *Rickettsia*
9639	M	g	Parvovirus; *Bartonella*; *Borrelia*	*Bartonella*; *Borrelia*		*Bartonella*	
9DCA	M	g	*Bartonella*; *Borrelia*	*Bartonella Borrelia*		*Bartonella*	
B877	F	a	*Y. enterocolitica*; *Bartonella*	*Bartonella*	*Bartonella*	*Y. enterocolitica*	
7FAO	M/j	a	*Bartonella*	*Bartonella*			
657C6	F	d	*Parvovirus*; *Borrelia*		*Parvovirus; Borrelia*		
J764	F/j	c	*S. enterica*; *Rickettsia*	*S. enterica*			*Rickettsia*
A123	M	e	*Orbivirus*; *Bartonella*	*Bartonella*	*Bartonella*		*Orbivirus*

*Hypothetical changes in pathogen load for each individual over time for 10 wild, urban rats, Rattus norvegicus, monitored over a minimum of 9 months to capture the widest possible range of temperature fluctuations. Target population should be 20 animals × 7 sites = 140 marked individuals. Δ, emergence of new pathogen. Incidence: Parvovirus 30%, Bartonella 70%, Borrelia 40%, Y. enterocolitica 20%, Orbivirus 20%, S. enterica 10%, Rickettsia 10%*.

**Table 3:** **Recruitment by pheromone-type**.

Rat	Sex/juvenile	Total visits	Pheromone type	Average visits/day	Sig (*T*; *P*)	Average dwell time ± (s)	Sig (*T*;*P*)
**By individual**							
J876	M	1072	Sebum	4.5		3.2	
M469	F	983	Sebum	2.8		2.0	
0B99	M	796	Sebum	1.1		1.6	
9639	M	557	Sebum	0.4		2	
9DCA	M	978	Sebum	2.5		0.5	
B877	F	690	Sebum	3.8		2.3	
7FAO	M/j	1235	Sebum	4.6		4.8	
657C6	F	787	Sebum	7.5		6.25	
**By sex**							
Male		846		2.6	34.7; **<0.05**	3.5	0.98; 0.245
Female		1011		4.7		2.4	

*Example recruitment to RFID sensor for wild, Norway rats, Rattus norvegicus, based on pheromone-type placed at sensor and monitored over 6 months. Target population should be 20 animals × 7 sites = 140 marked individuals. Pairwise comparisons of visitation or dwell times can be performed between different pheromone types × sex. Bold values indicate significance at the 0.05 level*.

## Discussion

Rat research is becoming more important for increasingly urbanized, and thus vulnerable, human populations. However, new methods are required to overcome the physical and social barriers that impede progress. In 2015, Firth et al. studied a population of only 133 rats in New York City, and discovered 18 pathogens previously unknown to science. Similarly, when the international Journal Vector-Borne and Zoonotic Diseases broadcasted a call for research in a special issue rodent-vectored pathogens and influence on human health, there were no scientific entries from North America (7). Clearly, new methodologies are required to address these significant barriers, and new detailed assays may help open the door for additional urban research.

We have shown that careful planning and following or modifying our protocol can yield a wealth of information regarding the ecology, time, and place of disease emergence and pathogenicity of wild rats in the urban environment. In a recent study (7), we used this protocol to determine what times animals were most active in winter, and segregated this information by sex and size/relative age, and assessed the health of individuals by noting how previously chipped-animals changed over time. This can be done by examining the body of recaptured animals (length of guard hairs, vibrissae, level of porphyrin), or by examining camera video or photographs for instances of new wounds and markings when the animal visits the sensor.

From an ethological perspective, we can deduce different behaviors between males and females, adults and juveniles, including dominant, subdominant behaviors, sexual receptivity, and peak activity times. Most importantly, the data will encode the individual identification of rodents that are assessed for pathogens, and importantly, of repeatedly captured individuals, so that changing pathogen loads can be monitored over time. For instance, we may find a higher incidence of arthropod-borne bacteria, such as *Borrelia* or *Rickettsia*, late in the season, whereas they may have been absent during the early spring. Additionally, some rodents may pickup additional pathogens as they seasonally migrate from public park spaces to the sewers in the winter before re-emerging and potential immigration (a process called “vertical migration,” Corrigan, personal communication).

While methodology such as this requires significant preparation and pre-planning of both laboratory and field gear, we note that the overall cost of our purchases was less than $15,000 USD; an insignificant cost considering the implications on our health and estimated $19 billion per annum that rats cost society (3). Potential modifications to our protocol might include use of a subset of chemicals in the mixed pooled scents, such as androgens to attract dominant males, or MUPs to attract adult females, potentially in the absence of males. The latter approach might be particularly relevant for the increased trapping of adult females into single-use, injectable immunocontraceptive traps. While we cannot fully overcome the social “taboo” nature of working with wild rats, open communications followed by new methodology and approaches may help reduce the physical barriers that are compounded by the social anxieties.

## Notes

The protocols we recommend are sufficient to provide serum and rectal fluids necessary for targeted molecular analysis and UHTS assays. However, we note that tissue distribution of viruses is not possible without sacrificing the animal. Thus, our assay is intended to enable the documentation of changes in known microbial diversity and distribution, as well as viral incidence, and not to determine the tissue-level sites of viral replication (within the animal).

## Ethics Statement

All procedures are in accordance with the guidelines for ethical conduct in the care and use of non-human animals in research (Hofstra IACUC #0093).

## Author Contributions

MP and MD conceived and designed the protocol. MP wrote the manuscript with editorial input from RS and MD.

## Conflict of Interest Statement

The authors declare that the research was conducted in the absence of any commercial or financial relationships that could be construed as a potential conflict of interest.
