# Supplementary material for: A Detailed Protocol to Enable Safe-Handling, Preemptive Detection, and Systematic Surveillance of Rat-Vectored Pathogens in the Urban Environment
Source: Front Public Health. 2016 Jul 14;4:132. doi: 10.3389/fpubh.2016.00132 (PMC4945852; doi:10.3389/fpubh.2016.00132)
Supplement: Supplementary file 1 [file Image_1.PDF]

Look, Listen and Learn: Signs of rat presence

Look for:

- Rat droppings
- Gnawing
- Burrows
- Runways
- Tracks
- Grease marks (sebum)
- Urine stains or smells
- Visual sightings of rats

Listen for:

- Rodent sounds

Learn where:

- Rubbish/wastes are accumulated
- Rat entry points

Fresh urine will glow yellow, old urine will have a blue tint under UV backlight (365-395 nm).
Report unusual sightings to your local health department.

Figure S1. "Look, listen and learn" scoping procedure for pest management professionals (PMP) to identify signs associated with rat infestation. You may print and laminate this table as a stand-alone and distribute to students or members of the community.
